# Comparative isoenzyme electrophoreses between the brown-spotted locust, *Cyrtacanthacris tatarica* , and the desert locust, *Schistocerca gregaria*

**DOI:** 10.1093/jis/14.1.20

**Published:** 2014-01-01

**Authors:** G. Elsayed, S. A. M. Amer

**Affiliations:** 1 Faculty of Science, Taif University, P.O. 888, Taif, Kingdom of Saudi Arabia; 2 Department of Economic Entomology, Faculty of agriculture, Cairo University, Egypt; 3 Department of Zoology, Faculty of Science, Cairo University, Egypt

**Keywords:** gene locust, guardian locust, Saudi Arabia

## Abstract

The desert locust, *Schistocerca gregaria* (Forskål) (Orthoptera: Acrididae), and the brown-spotted locust, *Cyrtacanthacris tatarica* (Linné) (Orthoptera: Acrididae), were collected from Saudi Arabia to investigate their relationships *.* Native polyacrylamide gel electrophoreses of five arbitrarily chosen metabolic enzymes extracted from the leg muscles of the two locust taxa were conducted. These enzymes were acid phosphatase ( *Acph* ), alcohol dehydrogenase ( *Adh* ), βester-ase ( *β est* ), malic enzyme ( *Mal)* and malate dehydrogenase ( *Mdh* ). Twenty presumptive gene loci and 26 polymorphic alleles were recorded. *Acph* did not discriminate between the two locust species, while the other four isoenzymes discriminated between them. Most of the alleles were monomeric, but *Mal* and *Mdh* exhibited dimeric alleles in the samples of *C. tatarica* . *βest* frac-tions were more expressed in *C. tatarica* , and the three enzymes *βest* , *Mal* , and *Mdh* discriminated clearly between the two species. The similarity coefficient that was calculated according to the number of sharing alleles between the two locusts was found to be 0.69. The isoenzyme variation presented herein seemed to reflect either their physiological adaptation or the taxonomic consequences between the two taxa. Collecting more isoenzymes for more sam-ples could have taxonomic value.

## Introduction

The desert gregarious locust, *Schistocerca gregaria* (Forskål) (Orthoptera: Acrididae), is the only representative of its genus in the old world ( Song 2004 ). The brown-spotted locust, *Cyrtacanthacris tatarica* (Linné) (Orthoptera: Acrididae), is a solitarian guardian locust found in the scattered vegetation of grasses, herbs, and shrubs in west Asia ( Suhail et al. 2001 ). The two grasshoppers belong to the subfamily Cyrtacanthacridinae and family Acrididae. The desert locust is infamous for forming enormous swarms and it annually causes severe agricultural and economic damage ( Uvarov 1966 , 1977; Showler 1995 ; Pener and Yerushalmi 1998 ). In Saudi Arabia, the two species were shown to have two different phases: the gregarious phase is for *S. gregaria* and the solitarian phase is for *C. tatarica* .

Chromosomal and molecular markers were used to investigate the relationships for species and populations of Acridoidea ( Xuan et al. 2003 ). Few isoenzymatic studies have been applied for populations and species of Acridoidea ( Moran et al. 1980 ; Gill 1981 ; Halliday et al. 1983 ; Chapco and Bidochka 1986 ). Recently, several allozymic and molecular studies on locusts and grasshoppers ( Zheng et al. 2006 ; Chapuis et al. 2008 ; Zhang et al. 2009 ; Gu et al. 2010 ; Li et al. 2010 ) were reported with no reference to the Saudi Arabian desert locust. Therefore, our study was conducted to: a) detect specific gene loci for the two locusts; b) determine the degree of differentiation between the two species; and c) correlate genetic and physiological variation.

## Materials and Methods

### Sample preparation and enzyme assay

Femur samples from 12 individuals from both locust species were collected randomly from Taif Governorate of Saudi Arabia. For isoenzyme extraction, approximately 0.5 g of tissue was homogenized in 1 mL saline solution NaCl (0.9%) using a manual homogenizer. The homogenates were centrifuged at 5000 rpm for 10 minutes and the supernatants were kept at -20ºC until use. Isoenzymes were elec-trophorased in 10% native-polyacrylamide gel as described by Stegemann et al. (1985) . For electrophoresis, 30 µL of the extract was mixed with 10 µL of treatment buffer and 35 µL of this mixture was applied to the well. After electrophoresis, the gels were stained according to their enzyme system with the appropriate substrate and chemical solutions, then incubated at room temperature in the dark for complete staining. In most cases, incubation for about one to two hours was enough.

### Isoenzyme assays

**Acid phosphatase(Acph).** After electrophoresis, the gel was soaked in 100 mL of 50 mM Na-acetate buffer with a pH of 5.0, containing 100 mg Fast Blue BB Salt (Sigma-Aldrich, www.sigmaaldrich.com ), 100 mg α-naphthyl phosphate, 100 mg MgCl2, and 100 mg MnCl2 ( Wendel and Weeden 1989 ).

**Alcohol dehydrogenase ( *Adh* ).** After electrophoresis, the gel was soaked in a solution of 4 mL isopropanol, 25 mg NAD, 20 mg NBT, and 5 mg PMS in 100 mL of 0.05 M Tris HCl with a pH of 8.5 ( Jonathan and Wendel 1990 ). 50 mM Na-acetate buffer, pH 5.0, was prepared by adding 5.15 mL glacial acetic acid and 2.85 g sodium hydroxide to 500 mL distilled water.

**βEsterase enzyme (β *est* ).** After electrophoresis, the gel was soaked in 0.5 M borate buffer (pH 4.1) for 90 minutes at 4°C. This procedure lowers the pH of the gel from 8.8 to about 7, at which the reaction proceeds readily. The low temperature minimizes diffusion of the protein within the gel. The gel then was rinsed rapidly in two changes of double distilled water. The gel was stained for esterase activity by incubation at 37°C in a solution of 100 mg β-naphthyl acetate (as a substrate) and 100 mg Fast Blue RR Salt (Sigma-Aldrich) in 200 mL of 0.1 M phosphate buffer (pH 6.5) ( Scandalios 1964 ).

**Malic enzyme ( *Mal* ).** After electrophoresis, the gel was soaked in 100 mL of 0.05 M Tris HCl with a pH of 8.5 containing 25 mg NBT, 25 mg EDTA, 25 mg NADP, 10 mg malic acid, 100 MgCl2, and PMS ( Jonathan and Wendel 1990 ).

**Malate dehydrogenase ( *Mdh* ).** After electrophoresis, the gel was soaked in 100 mL of 0.05 M Tris HCl (pH 8.5) containing 25 mg NBT, 25 mg EDTA, 25 mg NAD, 10 mg malic acid, and 3 mg PMS ( Jonathan and Wendel 1990 ). 0.05 M Tris HCl (pH 8.5) was prepared by dissolving 0.605 g Tris in 50 mL distilled water. The pH was adjusted to 8.5 by HCl. Then the solution was completed to 100 mL by distilled water.

### Gel fixation

After the appearance of the enzyme bands, the reaction was stopped by washing the gel two or three times with tap water. This was followed by adding the fixative solution, which consisted of ethanol and 20% glacial acetic acid (9:11 v/v). The gel was kept in the fixative solution for 24 hours and then was photographed. All gels were scanned using the Gel Doc-2001 Bio-Rad system ( www.bio-rad.com ). For isoenzymes, the bands of enzyme activity were designated using the known system of nomenclature ( Allendorf and Utter 1978 ). An abbreviation that corresponds to the name of the enzyme designated each locus. When multiple loci were involved, the fastest anodal protein band was designated as locus one, the next as locus two, and so on. The similarity coefficient was calculated according to Nei and Li (1979) and Lynch (1990) as follows: 2 (number of sharing bands) / number of bands in population A + number of bands in population B.

## Results and Discussion

**Figure 1:**
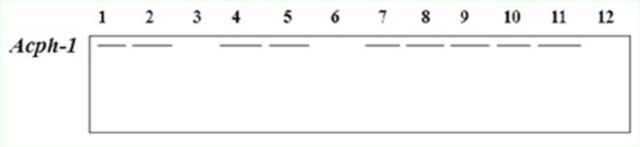
Electrophoretic banding pattern of *Acph* phenotypes for *Schistocerca gregaria* (1-6) and *Cyrtacanthacris tatarica* (7-12)

**Figure 2:**
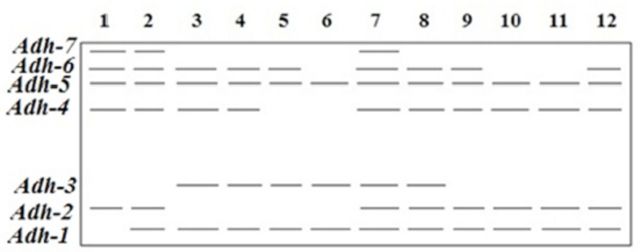
Electrophoretic banding pattern of *Adh* phenotypes for *Schistocerca gregaria* (1-6) and *Cyrtacanthacris tatarica* (7-12).

**Figure 3:**
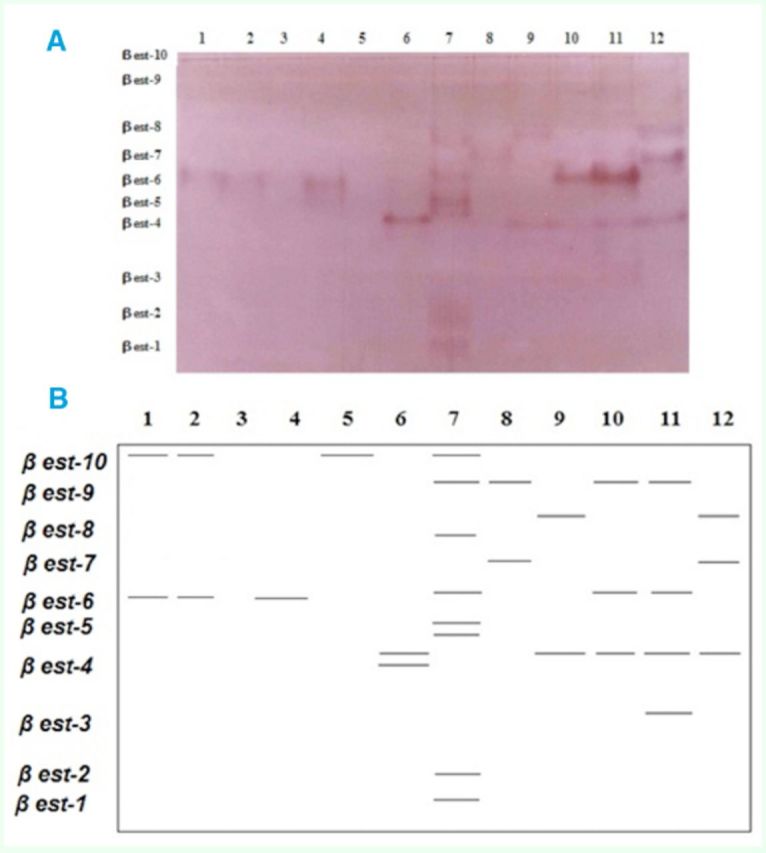
Electrophoretic banding pattern of *b est* phenotypes for *Schistocerca gregaria* (1-6) and *Cyrtacanthacris tatarica* (7-12). A) Original gel. B) Drawing of gel.

**Figure 4:**
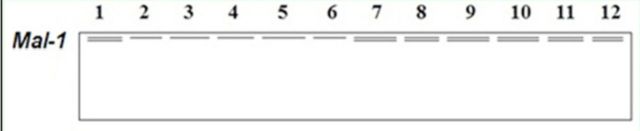
Electrophoretic banding pattern of *mal* phenotypes for *Schistocerca gregaria* (1-6) and *Cyrtacanthacris tatarica* (7-12).

**Figure 5.**
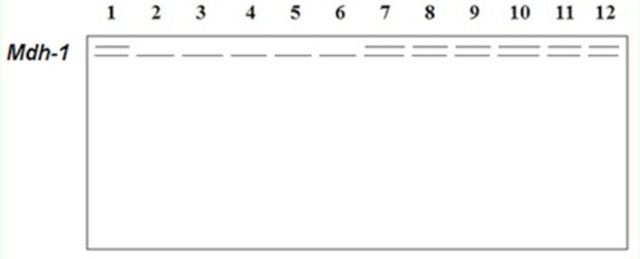
Electrophoretic banding pattern of *mdh* phenotypes for *Schistocerca gregaria* (1-6) and *Cyrtacanthacris tatarica* (7-12).

The allozymic patterns and alleleic variations of the studied isoenzymes are shown in Figures 1–5 . The five enzymes recorded a total of 20 polymorphic loci and 26 heterogeneous alleles. Electrophoresis of *Acph* isoenzyme showed monomorphic and fixed alleles in most studied samples of both species, with the same relative mobility near the top of the gel ( Figure 1 ). Two samples of *S. gregaria* and one sample of *C. tatarica* did not show any band.

For the *Adh* isoenzyme ( Figure 2 ), seven monomeric alleles were recorded, with the slowest allele ( *Adh-7* ) being less expressed, as it was recorded only in three samples. The loci *Adh-1* , *Adh-4* , and *Adh-5* were highly expressed (appeared in most samples studied). *Adh-2* appeared to be more expressed in *C. tatarica* (appeared in five out of six samples), while *Adh-3* was more expressed in *S. gregaria* (recorded in four out of six samples). *Adh-6* was nearly equally expressed in both species. Generally, this isoenzyme showed similar expression in both *C. tatarica* and *S. gregaria* .

The most variable allozymes were those of *βest,* where 10 phenotypes were produced ( Figure 3 ) in *C. tatarica* . In contrast, only *βest-4* , *βest-6* , and *βest-10* were recorded in *S. gregaria* , with *βest-4* appearing only in one sample showing double bands. Similar results for female *S. gregaria* were obtained by Rashad (2008) . *βest* , therefore, could be considered a good biochemical marker to discriminate clearly between the two locust species.

Esterases are classified as hydrolases, a large and diverse group of enzymes that catalyze the hydrolysis of a wide range of aliphatic and aromatic esters, choline esters, and organophosphorous compounds ( Dauterman 1985 ). Esterases act on molecules that are completely dissolved in water, hydrolyzing carboxylesterases into alcohol and carboxylate. They may break down cholesterol and are important in the resistance to insecticides and plant secondary substances ( Klowden 2007 ). The level of insect esterase is highly variable depending on the life stage, sex, tissue, hormones, breed, food, environmental conditions, and numerous other factors ( Devorshak and Roe 1999 ). The patterns of variation of this enzyme between the two species in our study could be attributed, therefore, to their difference in habitat, phenotyping, and whether they are solitarian or gregarious.

*Mal* showed one locus near the top of the gel with dimeric alleles in all samples of *C. tatarica* ( Figure 5 ) and only in one sample of *S. gregaria* . As this isoenzyme showed monomeric loci in most samples of *S. gregaria* and dimeric loci in all samples of *C. tatarica* , it could also be considered as a genetic marker to differentiate between the two locust species. Since *Mal* has an important role in bioenergetics ( Pon et al. 2011 ), it showed clear differences between both species in its pattern, as *S. gregaria* inhabits deserts while *C. tatarica* inhabits gardens.

*Mdh* isoenzyme recorded one monomeric isoform at the top of the gel in five out of six samples of *S. gregaria* . However, it showed dimeric locus in all samples of *C. tatarica* ( Figure 5 ) and, therefore, it could also be considered a good marker for differentiation between both species. Chan et al. (1991) revealed similar results for the grasshopper *Oxya japonica japonica* , where one of the two autosomal loci for *MDH* , the *Mdh-2* locus, controlling the anodal set of *MDH* isozymes, was duplicated.

Among the 26 recorded alleles, the number of common sharing alleles between the two species was nine alleles. The similarity coefficient that was calculated according to this number between the two species was found to be 0.69. Our study, therefore, revealed some differences between the two locusts on genetic and physiological levels. It would be necessary to analyze more isoenzymes for more samples to address this point more definitely.

## Abbreviations

*Acph*: acid phosphatase
*Adh*: alcohol dehydrogenase
*β est*: β esterase
*Mal*: malic enzyme
*Mdh*: malate dehydrogenase
